# Complex Management of Respiratory Failure, Atrial Fibrillation, Ventricular Tachycardia, and Stenotrophomonas maltophilia in a Patient Following Osteomyelitis Amputation: A Case of Multisystem Complications Occurring Postoperatively

**DOI:** 10.7759/cureus.73505

**Published:** 2024-11-12

**Authors:** Jesse O'Rorke, W. Greyson Butler, Keri Mason

**Affiliations:** 1 Medicine, Lee Health, Fort Myers, USA; 2 Medicine, Lake Erie College of Osteopathic Medicine, Bradenton, USA; 3 Internal Medicine, Cape Coral Hospital, Fort Myers, USA

**Keywords:** atrial fibrillation with rapid ventricular response, heart failure with preserved ejection fraction (hfpef), idiopathic ventricular tachycardia, mdr bacteria infection, osteomyelitis diagnosis, respiratory failure, severe respiratory failure, sodium-glucose cotransporter-2 (sglt2) inhibitors, stenotrophomonas maltophilia infection

## Abstract

*Stenotrophomonas maltophilia *is an opportunistic, multidrug-resistant gram-negative bacterium often affecting patients with significant comorbidities. This case report examines the hospital course of a 75-year-old male with a history of atrial fibrillation and heart failure with preserved ejection fraction (HFpEF), who presented with compromised respiratory status and recurrent infections, highlighting the complexities of clinical management in the setting of multidrug-resistant HFpEF organisms and postoperative complications.

The patient was admitted following an episode of ventricular tachycardia and acute respiratory failure, requiring rapid airway management and intensive clinical intervention. His recent hospitalization for sepsis, pneumonia, and osteomyelitis complicated his clinical profile, particularly given his recurrent urinary tract infections (UTIs), which prevented the use of sodium-glucose cotransporter-2 inhibitor therapy for heart failure management. Respiratory cultures confirmed the presence of *S. maltophilia*, prompting treatment with minocycline and piperacillin-tazobactam.

This case highlights the significant risks associated with postoperative arrhythmias in patients with underlying cardiac disease, particularly when superimposed with sepsis. Furthermore, the management of recurrent UTIs posed a barrier to optimizing heart failure therapy, further complicating the patient's clinical stability. The need for vigilant monitoring and tailored therapeutic strategies is essential to improve outcomes in this vulnerable patient population.

The interplay between multidrug-resistant infections, arrhythmias, and comorbidities emphasizes the importance of comprehensive clinical management and the need for further research to develop targeted therapies and clinical plans for at-risk populations.

## Introduction

*Stenotrophomonas maltophilia* is a multidrug-resistant gram-negative bacterium, which can cause serious infections in hospitalized patients, particularly those who are immunocompromised or who have recently undergone invasive procedures. Although relatively uncommon compared to other nosocomial pathogens, it is notable for its ability to cause severe respiratory complications, which can lead to high morbidity and mortality. This pathogen commonly colonizes the respiratory tract, causing life-threatening infections such as pneumonia and bacteremia. *S. maltophilia* most frequently infects mechanically ventilated patients; however, the use of indwelling catheters or central venous catheters can also lead to infection [1]. Additionally, recent use of broad-spectrum antibiotics further predisposes patients to colonization and infection by this resistant organism [2].

The treatment of *S. maltophilia *infections is challenging due to its resistance to most beta-lactams, fluoroquinolones, aminoglycosides, and macrolides, leaving limited therapeutic options. Among these, trimethoprim-sulfamethoxazole and minocycline have proven to be effective. In vulnerable patient populations, such as the one presented in this case, respiratory infections caused by *S. maltophilia* can further complicate recovery and clinical management, highlighting the importance of early detection and targeted therapy to optimize outcomes.

## Case presentation

A 75-year-old male presented to the emergency room with a chief complaint of shortness of breath and altered mental status. The emergency medical response team noted that the patient was hypoxic on arrival and that he was displaying signs of respiratory distress. During the ambulance ride to the emergency room, he experienced atrial fibrillation with a rapid ventricular response and episodes of ventricular tachycardia, which required two cardioversions and a dose of amiodarone. He was intubated en route to the emergency room for airway protection. This patient has a past medical history of atrial fibrillation, heart failure with preserved ejection fraction (HFpEF), alcoholic polyneuropathy, obstructive sleep apnea, hypertension, benign prostatic hyperplasia, deep venous thrombosis, and recurrent urinary tract infections (UTIs). Additional history includes hospitalization for six days and being discharged the day before this presentation. His last hospitalization was due to sepsis with concomitant pneumonia and right great toe osteomyelitis as identified in an outpatient MRI. 

**Figure 1:**
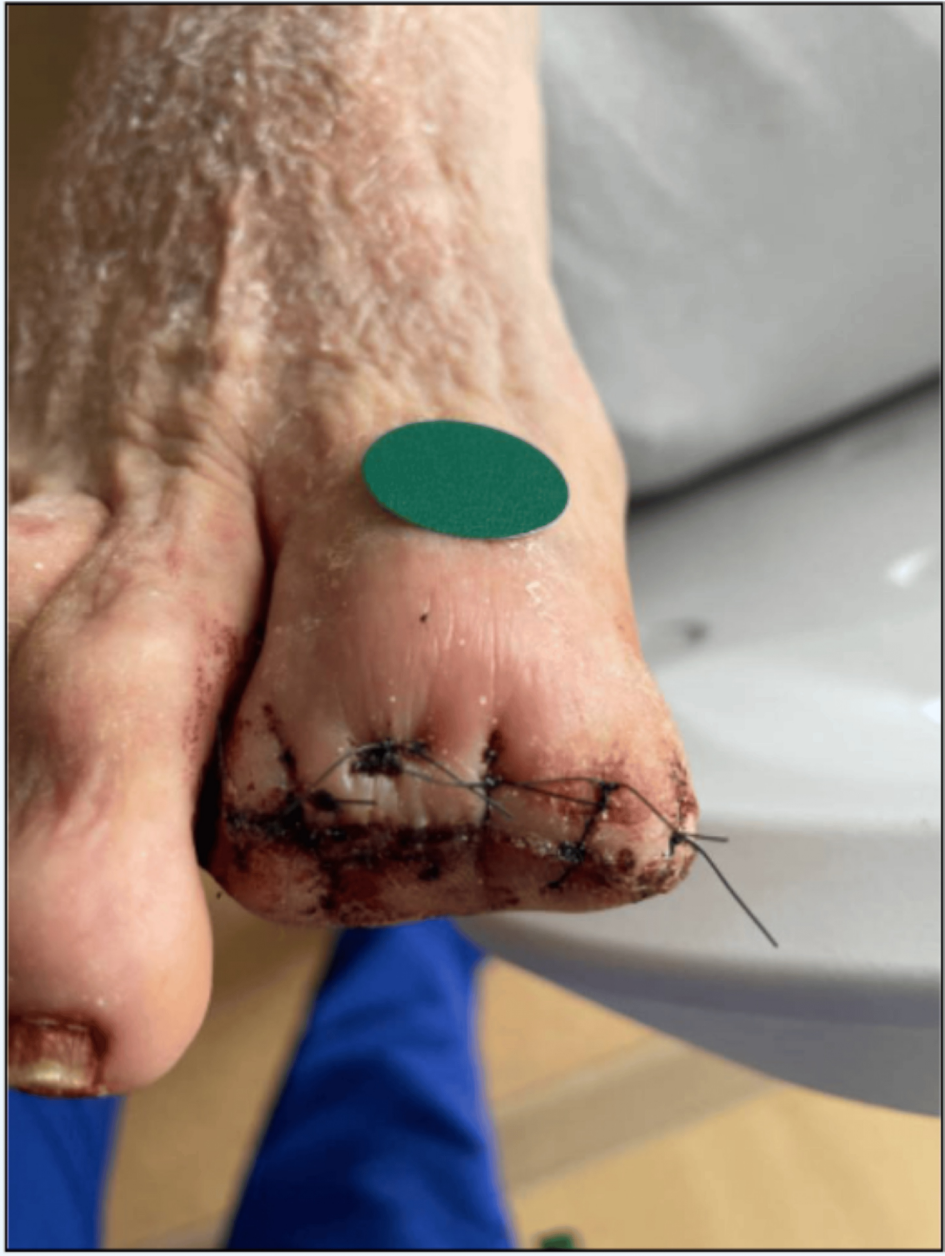
A photograph of the patient’s toe as taken on his prior admission, status post partial hallux amputation.

The patient reported that he was unclear on the etiology of the great toe osteomyelitis and that he had the issue for several months until he saw a wound care physician to have vascular studies and the MRI. He underwent partial hallux amputation during that hospitalization, a photograph taken post operation is shown in Figure 1. He was discharged in a clinically stable state with referrals to see an outpatient podiatrist and infectious disease doctor on a 12-day course of oral antibiotics, specifically amoxicillin-clavulanate and minocycline.

For the current hospitalization, the patient was admitted for ventricular tachycardia and acute respiratory failure. He was placed on IV amiodarone and IV heparin and noted to be hypotensive and started on intravenous fluid, Levophed, and vasopressin. An echocardiogram revealed a preserved ejection fraction of 50-55%, compared to a previous echocardiogram two months earlier that showed an ejection fraction of 55-60%. High-sensitivity cardiac troponin levels were also measured and showed no evidence of significant cardiac damage. Upon admission, he was started on IV piperacillin-tazobactam and minocycline due to osteomyelitis in his first toe and an expectorated sputum culture from his previous admission, which had grown *S. maltophilia* (Table 1).

**Table 1:** Respiratory culture results showed the growth of Stenotrophomonas maltophilia and confirmed the sensitivity of the organism to minocycline, which was used in the treatment of this patient. LPF, low-power field

Culture, respiratory, lower	
Respiratory culture	Numerous Stenotrophomonas maltophilia
WBC	<10 WBC’s/LPF
Epithelial cells	No epithelial cells/LPF
Gram stain	Few gram-negative rods
Stenotrophomonase maltophilia sensitivity	
Levofloxacin	Resistant
Minocycline	Sensitive
Trimethoprim/sulfamethoxazole	Sensitive

**Figure 2:**
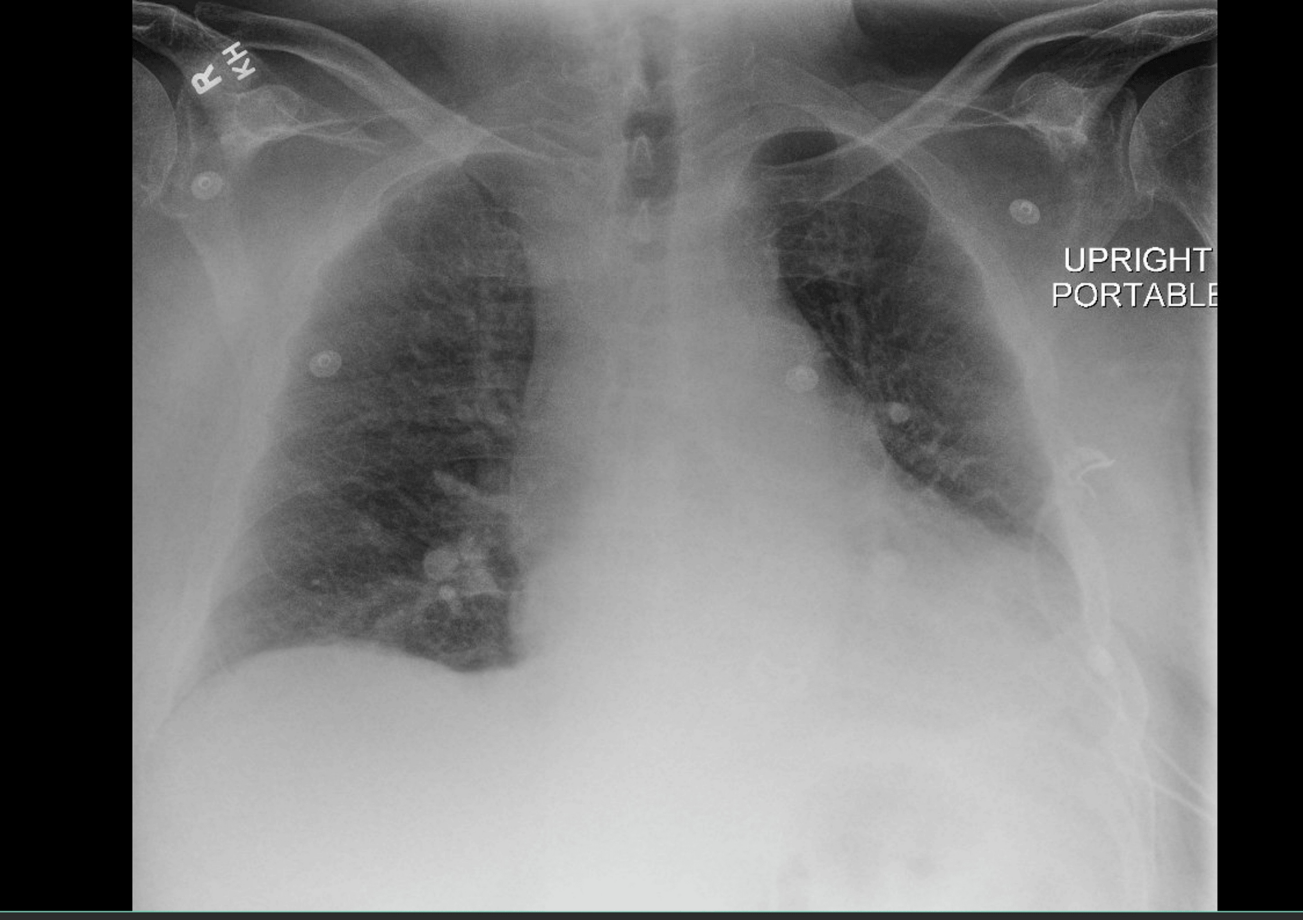
Patient’s chest radiograph significant for cardiomegaly, pulmonary edema, bilateral interstitial infiltrates, patchy airspace opacities, and bilateral pleural effusion.

At this point, the cardiology, pulmonology, infectious disease, and podiatry services were consulted. The patient was deemed to be a candidate for extubation on his second day of hospitalization and was successfully extubated and weaned to 4L of high-flow oxygen via nasal cannula. Vasopressors were also discontinued at this time. He was transitioned from IV heparin to his home dose of apixaban, and his blood pressure returned to normal. He was transitioned from IV amiodarone to 120 mg of diltiazem daily and 75 mg of metoprolol twice daily. He was noted to have left upper extremity swelling and bilateral lower extremity swelling. A venous duplex of his bilateral lower extremities was performed and showed acute superficial vein phlebitis of the bilateral great saphenous veins, with no evidence of a deep vein thrombosis. A venous duplex of the left upper extremity also showed no evidence of deep vein thrombosis. The patient was transferred out of the ICU at this time. Upon admission to the medical floor, he developed increasing shortness of breath. Chest X-ray was performed that was significant for increasing cardiomegaly, pulmonary edema, bilateral interstitial infiltrates, and patchy airspace opacities, with a bilateral pleural effusion (Figure 2)*. *He was started on IV Lasix for his volume overloaded state. He achieved significant diuresis, with 10 L of urine output and a decrease in his weight from 315 pounds to 289 pounds.

While in the hospital, the patient was noted to have a significant left sided weakness. MRI of his head showed no acute stroke, and bilateral carotid artery duplex was negative for any hemodynamically significant stenosis in the bilateral internal carotid arteries. A cervical MRI was performed that showed no evidence of spinal cord compression. The podiatry service evaluated the patient and reported that there was no recommendation for further surgical intervention. 

After 12 more rather unremarkable hospital days, the patient was deemed clinically stable for discharge. The patient completed his antibiotic regimen of 7 days of piperacillin/tazobactam then 3 days of amoxicillin/clavulanic acid and 10 days of minocycline, as indicated by cultures and prescribed and monitored by the infectious disease service. On discharge, the cardiology service increased his metoprolol to 75 mg twice daily for better heart rate control and cleared him as stable for discharge from a cardiovascular perspective. Physical therapy evaluated the patient and recommended placement in a skilled nursing facility, which the patient agreed to. While the patient was using oxygen during his hospitalization, he was able to be weaned and saturated well on room air for discharge. The patient was stable at discharge with the recommendation to follow-up as an outpatient with a podiatrist and his primary care physician. He was sent home with the recommendation to continue 5 mg of apixaban twice daily, 1% topical diclofenac four times daily, 100 mg of docusate sodium once daily, 5 mg of finasteride once daily, 2 L/minute of home oxygen, fluticasone-umeclidinium-vilanterol 100-62.5-25 mg inhaler one puff daily, 400 mg of magnesium oxide once daily, 100 mg of nitrofurantoin once daily for 90 days, 10 mEq of potassium chloride twice daily, 40 mg of rosuvastatin once daily, 8.6-50 mg of senna-docusate once nightly, and 10 mg of torsemide once every morning. 

## Discussion

**Figure 3:**
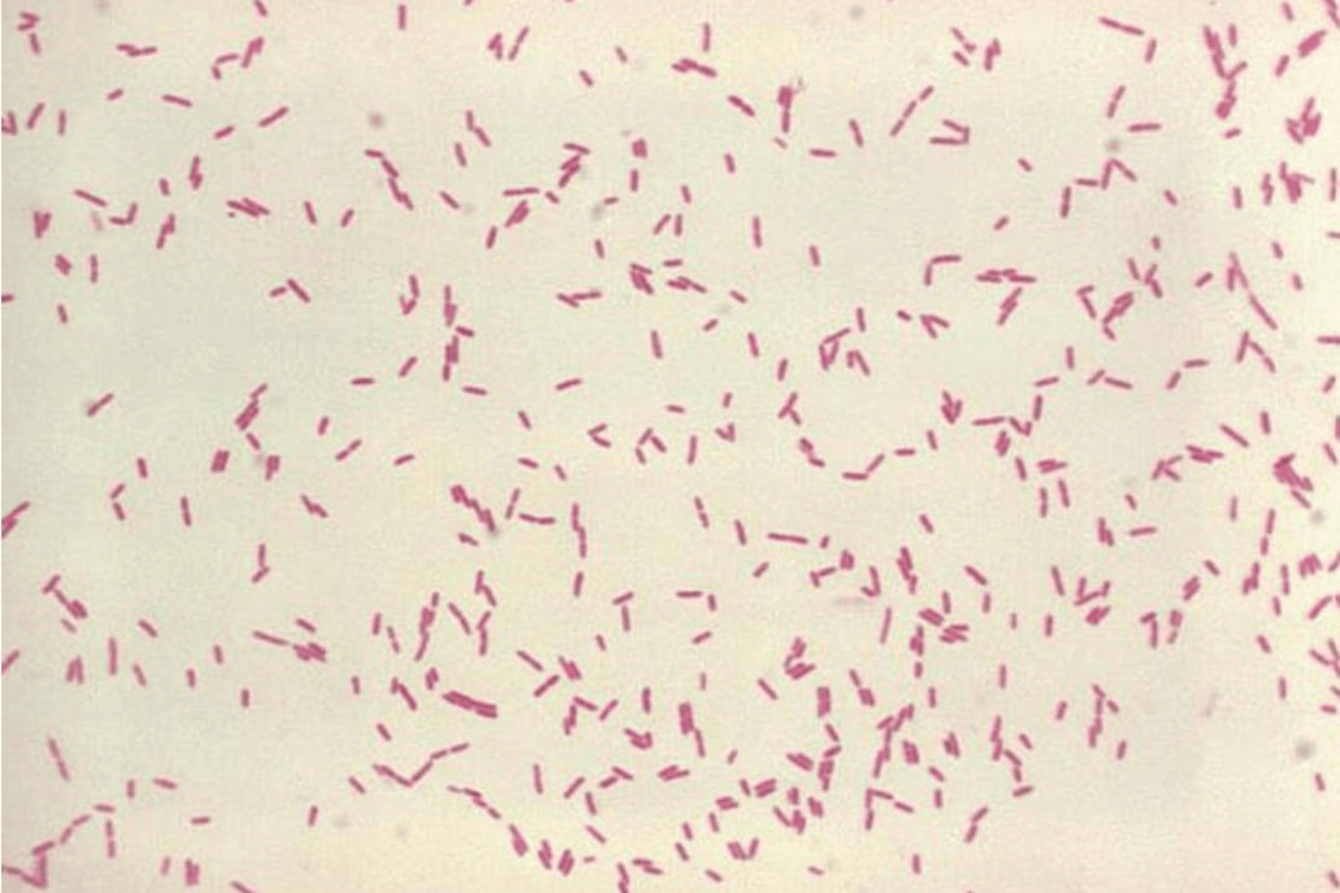
Illustration of a Gram stain of Stenotrophomonas maltophilia. Source: [9]

*Stenotrophomonas maltophilia* is an opportunistic, multidrug-resistant gram-negative bacterium that thrives in immunocompromised hosts or those who have undergone invasive procedures with Gram stain (Figure 3). It possesses a natural ability to attach to foreign surfaces and develop a biofilm, which provides it with protection against both the host immune response and antimicrobial treatments. This behavior is facilitated by factors such as its positively charged surface and the presence of fimbrial adhesins [3]. Additionally, several different mechanisms contribute to its antibiotic resistance. It produces two inducible beta-lactamases, a zinc-dependent penicillinase and a cephalosporinase, both of them play a role in its resistance to beta-lactams, carbapenems, and aztreonam by cleaving the beta-lactam ring of these antibiotics [4,5]. Resistance to aminoglycosides is driven primarily by an acetyltransferase, which acetylates the amino group of the aminoglycoside molecule typically at the 2’ or 6’-position of the drug, rendering it inactive [6]. Efflux pumps contribute to resistance to many other antimicrobials as well [7,8].

Due to the multidrug resistance exhibited by *S. maltophilia*, minocycline is considered one of the best treatment options. Minocycline has broad-spectrum antibacterial activity and is effective against many multidrug-resistant organisms. It is a tetracycline derivative and has shown excellent in vitro activity against *S. maltophilia* primarily due to its ability to inhibit protein synthesis by binding to the 30S ribosomal subunit [10]. It also has good tissue penetration and a long half-life, allowing for sustained antibacterial activity [11]. In addition, minocycline’s ability to bypass common resistance mechanisms, such as efflux pumps and beta-lactamase production, makes it particularly effective in treating *S. maltophilia* infection. Its efficacy, tolerability, and availability in both oral and intravenous forms make it a preferred drug in managing these difficult-to-treat infections. 

In addition to the significant respiratory effects an infection by *S. maltophilia* can have on a patient, septic conditions can significantly exacerbate cardiac arrhythmias, particularly atrial fibrillation with rapid ventricular response and ventricular tachycardia, contributing to a cycle of worsening cardiac and respiratory instability. Sepsis triggers a systemic inflammatory response that releases pro-inflammatory cytokines, causing widespread vasodilation, increased capillary permeability, and hypotension [12]. This inflammatory cascade can place additional stress on the cardiovascular system, particularly in patients with pre-existing heart conditions. In such cases, the systemic effects of sepsis may disrupt the balance between preload and afterload, precipitating episodes of arrhythmia such as atrial fibrillation with rapid ventricular response. 

The inflammatory response associated with sepsis can also directly irritate the myocardium, creating electrical instability within the atria and promoting rapid, irregular heart rhythms [13]. The elevated heart rate seen in atrial fibrillation with rapid ventricular response places an additional burden on the failing heart, impairing ventricular filling and reducing cardiac output, particularly in those with HFpEF. A decreased cardiac output can lead to hypotension, poor perfusion, and worsening oxygen delivery to vital organs, further complicating respiratory status. 

**Figure 4:**
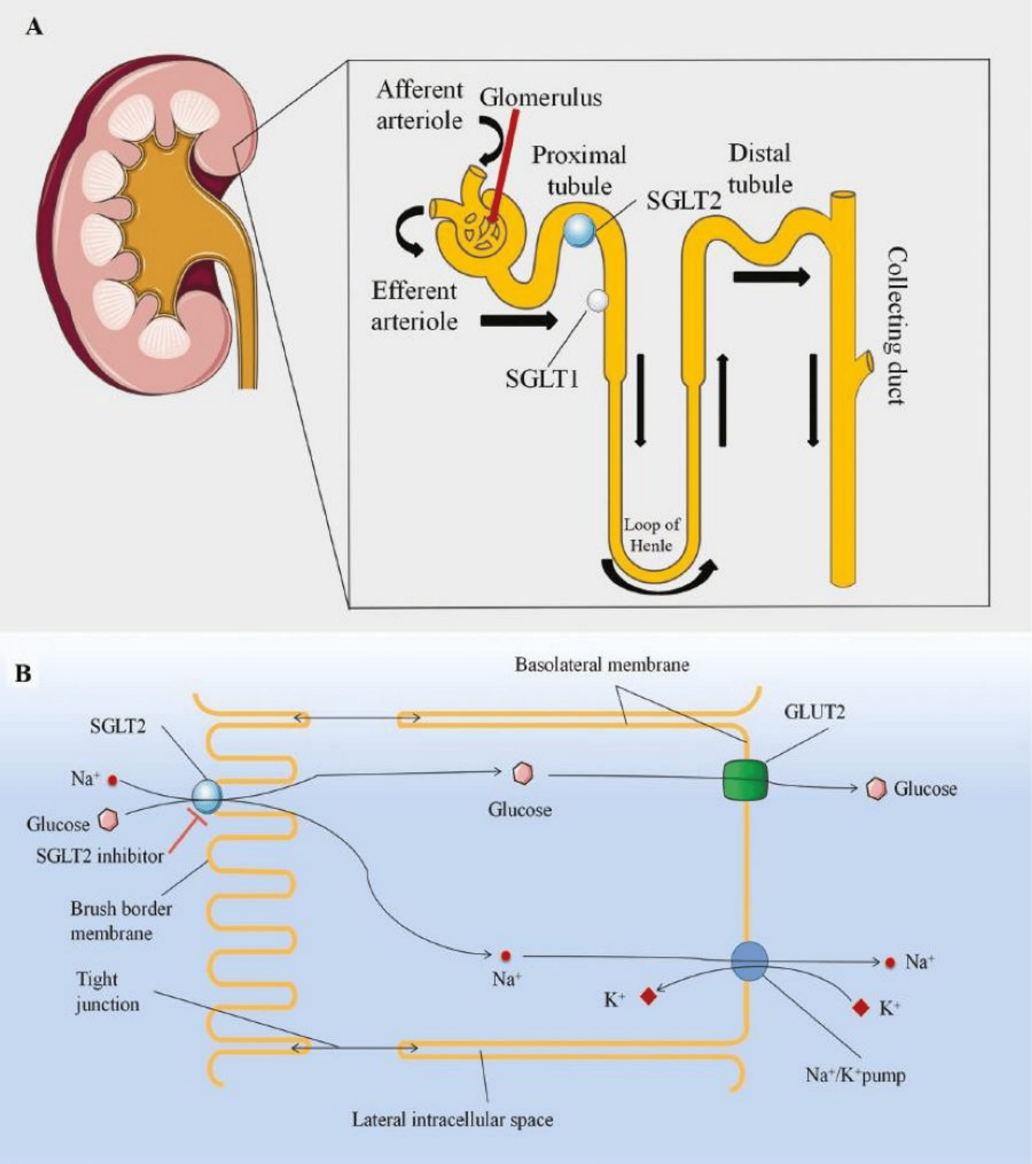
Illustration of the mechanism of action of SGLT2 inhibitors in the proximal convoluted tubule. Source: [16] GLUT2, glucose transporter 2; SGLT2, sodium-glucose cotransporter-2

While several medications are indicated for treating heart failure, sodium-glucose cotransporter-2 (SGLT2) inhibitors are widely regarded as part of the group of medicines used as first-line therapy. SGLT2 inhibitors work by blocking the SGLT2 in the kidneys, leading to increased urinary glucose excretion (Figure 4) [14]. This leads to osmotic diuresis, which decreases intravascular volume and reduces blood pressure and preload, easing the workload on the heart. This elevated glucose concentration in the urine also creates an environment conducive to bacterial growth, thereby increasing the risk of UTIs [15]. Because of this increased risk, patients with HFpEF may not be suitable candidates for SGLT2 inhibitors if they have a history of recurrent UTIs. For patients with a history of recurrent UTIs, this side effect complicates the management of HFpEF, as these infections can worsen overall health, trigger hospitalizations, and limit the use of an otherwise beneficial class of drugs.

## Conclusions

This case illustrates the complex interplay of multiple medical conditions and the significant challenges encountered in managing a postoperative patient with *S. maltophilia* infection, atrial fibrillation, and episodic ventricular tachycardia. The patient’s history of HFpEF and recurrent UTIs complicated the clinical landscape, necessitating a multidisciplinary approach to achieve stabilization. It is postulated that this patient’s cascade of medical complications in this particular episode could have been set off by the osteomyelitis in his great toe that spiraled into sepsis and respiratory collapse. This patient’s specific presentation with the rare microbe *S. maltophilia* further complicated measures, as extended course antibiotics needed to be started. 

This case perfectly represents how an infection that is “ignored” or put off by the patient can lead to nefarious complications down the road. The patient reported on admission that he had the toe wound for some months and that it had worsened to the point where he finally decided to seek treatment. Had that wound been identified and properly treated earlier, this clinical picture could have been very different. This problem of a lack of prompt identification and treatment can be further exacerbated in the population of patients with advanced diabetes mellitus with secondary peripheral neuropathy and those with peripheral neuropathy stemming from another etiology. We hope this literature can further emphasize the need for primary care physicians to perform a proper foot examination during routine checkups in patients who are deemed to be at higher risk for such wounds and infections. 

Moreover, this case presentation highlights the necessity for ongoing research into effective management strategies and targeted therapies for infections caused by resistant organisms. As healthcare providers encounter an increasing prevalence of multidrug-resistant bacteria, understanding the underlying mechanisms of resistance and developing tailored therapeutic approaches will be vital to improving patient outcomes in similar clinical scenarios. This case also serves as a reminder of the critical need for comprehensive care that addresses not only the immediate medical concerns but also the broader context of a patient’s health status and medical history. Through this report, we hope to contribute to the growing body of literature on the multimodal treatment necessary for patients with infections superimposed on chronic illness and provide insight that may be helpful in the management and prevention of similar cases in the future.
